# Flood hazard mapping and assessment in data-scarce Nyaungdon area, Myanmar

**DOI:** 10.1371/journal.pone.0224558

**Published:** 2019-11-26

**Authors:** Zaw Myo Khaing, Ke Zhang, Hisaya Sawano, Badri Bhakra Shrestha, Takahiro Sayama, Kazuhiro Nakamura

**Affiliations:** 1 State Key Laboratory of Hydrology-Water Resources and Hydraulic Engineering and College of Hydrology and Water Resources, Hohai University, Nanjing, Jiangsu Province, China; 2 CMA-HHU Joint Laboratory for HydroMeteorological Studies, Hohai University, Nanjing, Jiangsu Province, China; 3 Department of Meteorology and Hydrology, Ministry of Transport and Communications, Nay Pyi Taw, Myanmar; 4 International Centre for Water Hazard and Risk Management, Tsukuba-shi, Ibaraki-Ken, Japan; 5 Disaster Prevention Research Institute, Kyoto University, Gokasho, Uji, Kyoto, Japan; 6 Construction Technique Institute, Chuo-Ku, Tokyo, Japan; Bristol University/Remote Sensing Solutions Inc., UNITED STATES

## Abstract

Torrential and long-lasting rainfall often causes long-duration floods in flat and lowland areas in data-scarce Nyaungdon Area of Myanmar, imposing large threats to local people and their livelihoods. As historical hydrological observations and surveys on the impact of floods are very limited, flood hazard assessment and mapping are still lacked in this region, making it hard to design and implement effective flood protection measures. This study mainly focuses on evaluating the predicative capability of a 2D coupled hydrology-inundation model, namely the Rainfall-Runoff-Inundation (RRI) model, using ground observations and satellite remote sensing, and applying the RRI model to produce a flood hazard map for hazard assessment in Nyaungdon Area. Topography, land cover, and precipitation are used to drive the RRI model to simulate the spatial extent of flooding. Satellite images from Moderate Resolution Imaging Spectroradiometer (MODIS) and the Phased Array type L-band Synthetic Aperture Radar-2 onboard Advanced Land Observing Satellite-2 (ALOS-2 ALOS-2/PALSAR-2) are used to validate the modeled potential inundation areas. Model validation through comparisons with the streamflow observations and satellite inundation images shows that the RRI model can realistically capture the flow processes (R^2^ ≥ 0.87; NSE ≥ 0.60) and associated inundated areas (success index ≥ 0.66) of the historical extreme events. The resultant flood hazard map clearly highlights the areas with high levels of risks and provides a valuable tool for the design and implementation of future flood control and mitigation measures.

## Introduction

Due to rapid socioeconomic development and associated intensifying anthropogenic activities along with climate changes, losses of human lives and properties resulting from floods are increasing in many Asian countries in recent decades [1–4]. Heavy rainfalls due to prevailing monsoons or low-pressure systems (depressions and cyclones) and land use changes resulting from urban development favor the occurrence of high-magnitude floods in South Asia [5–7].

Myanmar is one of the South Asian countries that have frequently suffered from severe flood hazards. According to the historical records and flood mark surveys of the Department of Meteorology and Hydrology (DMH), Myanmar, the major flood years in recent decades include 1974, 1991, 1997, 2006, 2010, 2011, 2015 and 2016 [8]. In 2015, Myanmar experienced the most severe flooding in decades by unusually heavy monsoon precipitation that started in mid-July and continued to August [9, 10]. National Natural Disaster Management Committee (NNDMC) reported that the 2015 floods cost the lives of 120 people, forced over 1,624,000 people to be displaced, and inundated more than 500,000 ha of farmlands. According to the Agriculture and Livelihood Flood Impact Assessment, the floods severely affected the agricultural lands in six regions, especially in Nyaungdon Area that has more than 100,000 ha of land flooded.

Flooding in Nyaungdon Area, Myanmar is mainly a consequence of floodplain flooding. Flood duration and geographic extent of floods in Nyaungdon Area mostly depend on the influences of the Ayeyarwady River and its tributaries such as the Panhlaing River and Bawle River [8, 11]. In addition climate change induced extreme weather is likely to impose significant effects on hydrology and water resources of this area [12]. In this area, flood information like flood warnings and bulletins are unavailable due to lack of hydrometeorological data. As flood hazard maps provide the flood information including flood affected area and flood depth, the people who live in vulnerable areas can apply this information for proper flood protection measures. Unfortunately, flood hazard maps and other information such as soil erosion, river channel deformation, and land use/cover changes are generally lacked in Nyaungdon Area, making it even harder to design the flood mitigation plan in this region [13, 14].

Flood inundation mapping has played as an important role for designing sustainable urban plans, protecting human properties and lives, and mitigating disaster risks [15–17]. It is also a key step to develop a flood hazard map and conduct the proper flood assessment [18, 19]. Flood inundation mapping usually require repeated observations of flooded area and inundation extents through remote sensing images [20] or ground observations [21]. For data-scarce areas, hydrological and inundations models also play a critical role on flood simulations and risk assessment [4, 22, 23].

To gain the flood inundation and hazard maps in Nyaungdon Area, Myanmar, we applied a distributed, coupled hydrology-inundation model named the Rainfall-Runoff-Inundation (RRI) model to simultaneously simulate rainfall-runoff processes and flood inundation in Nyaungdon Area and to further derive the flood hazard map. This study aims to fill the knowledge and technical gap in the development of flood protection measures and flood risk management in the data-scarce Nyaungdon Area, Myanmar. The objectives of this study are two-fold: (1) to evaluate the predictive capacity of the RRI model in Nyaungdon Area, Myanmar, by comparisons with streamflow observations and satellite inundation images, and (2) to derive the flood hazard map in this region for further designing flood mitigation and defense plans in this region. This study presents a methodology based on topography, land cover, precipitation to calibrate the RRI model, simulate the spatial extent of flooding, and validate the potential flood inundated areas using satellite images. In this study, we aim to address two scientific questions: (1) how will the coupled hydrology-inundation RRI model and statistical methods perform and carry out to attain proper flood assessment in a data scarce area? and (2) can the developed model-based flood assessment fill the knowledge and technical gap in the development of flood protection measures and flood risk management?

## Materials and methods

### Description of the study area

**Fig 1:**
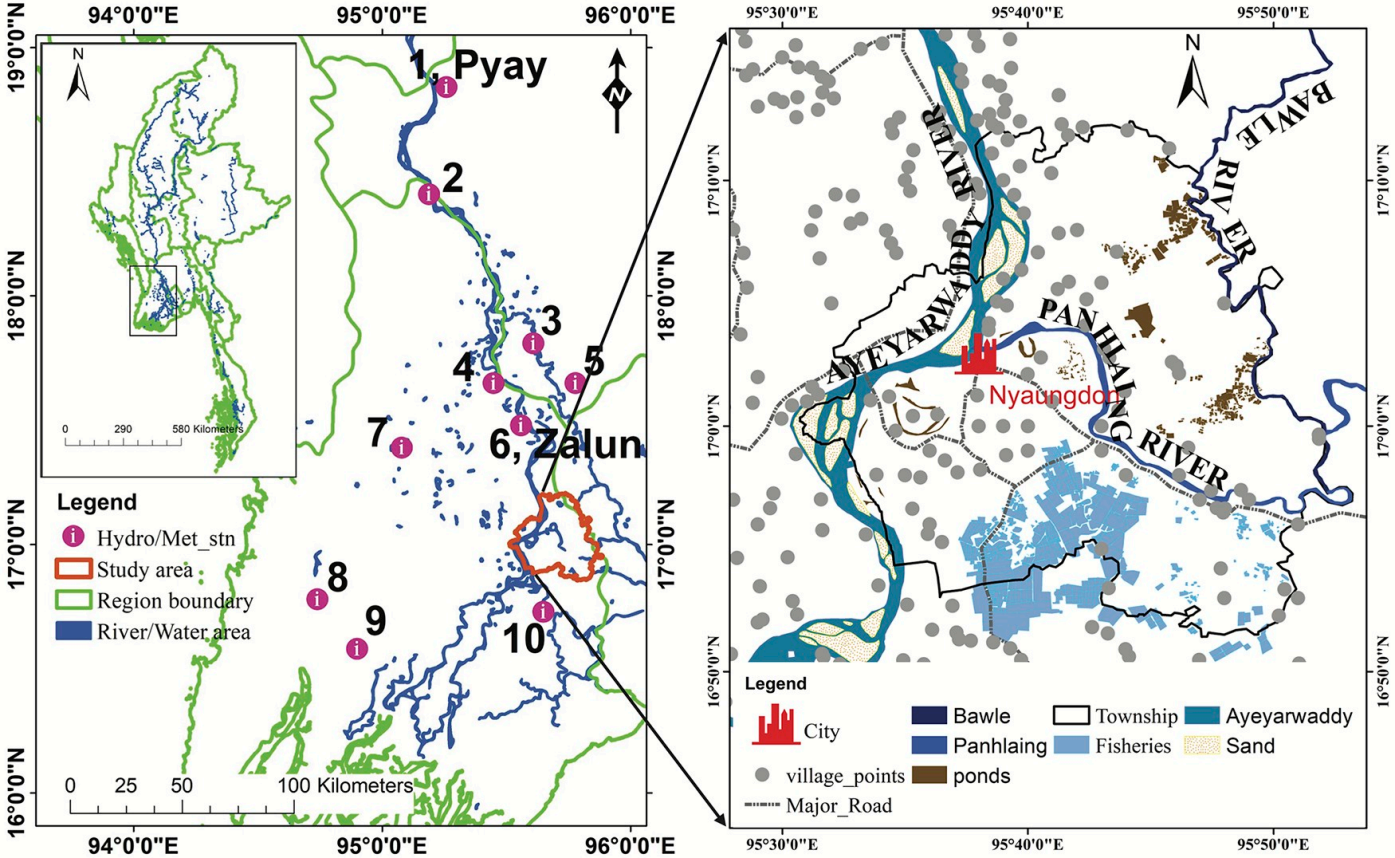
Locations of study area and the hydrological and meteorological observation stations.

Nyaungdon Township (NDT) is a township of the Maubin District of Ayeyarwady Region in Southern Myanmar (16°54´–16°59´N, 95°30´–95°45´E) and has a total area of ~899 km^2^. Fig 1 shows the locations of the hydrological and meteorological observation stations and the boundary of NDT. The study area and its surrounding area are flattening and floodplain zones [8]. According to the 15 arc-second (~450 m) digital elevation model (DEM) from the United States Geological Survey (USGS) HydroSHED (https://hydrosheds.cr.usgs.gov), elevation in the study area ranges from 2 to 44 m. As the weather condition of South West Monsoon favors precipitation in Myanmar, the area receives a large amount of rainfall each year [9, 24]. According to the long-term (1981~2010) meteorological records of the Ayeyarwady Region, annual rainfall is around 2500 mm, while annual maximum and minimum temperatures are ~37.0°C in April and ~15.0°C in January, respectively. Three rivers, namely Ayeyarwady, Panhlaing, and Bawle, flow through Nyaungdon Area (Fig 1). Normally, the flow of the Panhlaing River is about 25% that of the Ayeyarwady River.

**Fig 2:**
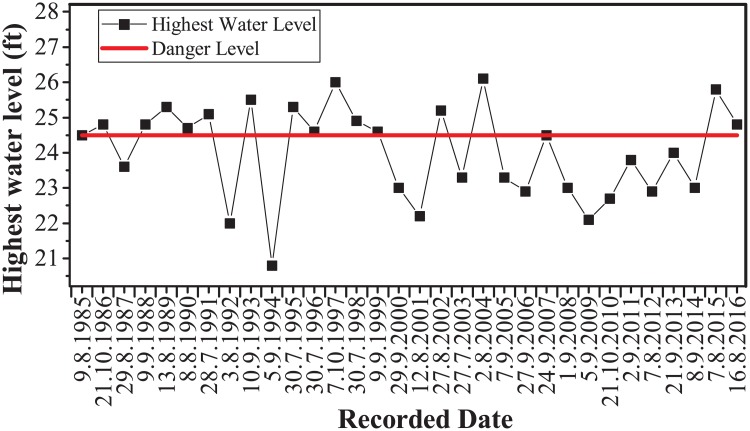
Records of annual highest water level of Panhlaing River at the Nyaungdon station from 1985 to 2016.

According to the records from Irrigation and Water Utilization Management Department (IWUMD), this area has frequently suffered riverine and widespread floods. For example, the water level of NDT at Panhlaing River reached above its danger level 15 times from 1985 to 2015 (Fig 2). The heavy rain linked to cyclone Komen in monsoon season of 2015 caused water-related disasters in many townships of Ayeyarwady Region, including NDT. According to the flood report of NNDMC (Sep 2015), the floods affected 19,076 acres of farmlands in NDT.

### Data sets and data sources

**Fig 3:**
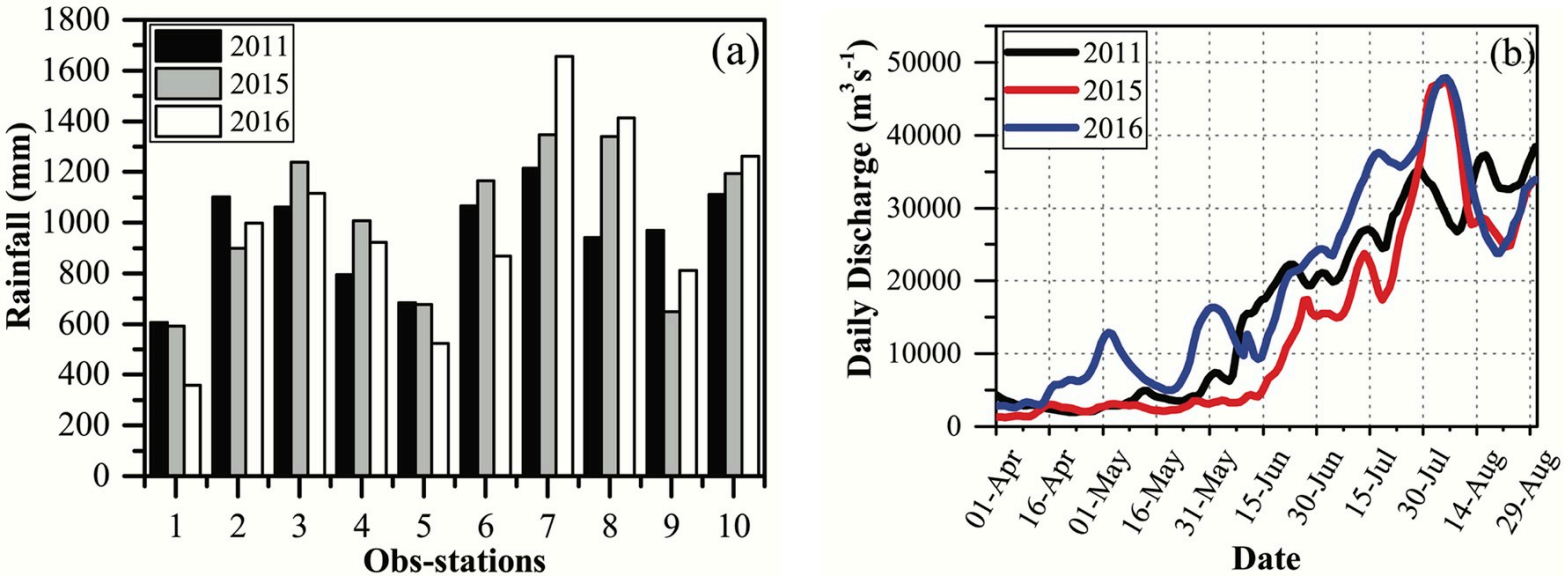
(a) Total rainfall hyetographs of ten observation stations and (b) hydrographs of upstream inflow observed at the Zalun station from April 1 to August 31 in 2011, 2015, and 2016.

There are a variety of data sets used for driving, calibrating and validating the RRI model in this study. Data sets used in this study were provided by or downloaded from relevant sources that are summarized in Table 1. Meteorological and hydrological data were collected from local government offices. Fig 3 shows the total rainfall hyetographs of ten rainfall stations and the hydrograph at the Zalun station located close to the upper boundary of the study area from April 1 to August 31 in 2011, 2015, and 2016. To assist the flood assessment and flood hazard mapping, the shapefiles (e.g. rivers, tributaries, built-up areas, ponds, swamps, roads, etc.) needed by the flood hazard map were digitized from the 2008 edition of Myanmar topography map in this study (see S3 Dataset). In this study, we were able to gain two sets of satellite images to validate the predicted flood extent areas of the 2015 and 2016 flood events. The observed flooded areas of the 2015 flood event were obtained the modified satellite image observed by the Phased Array type L-band Synthetic Aperture Radar-2 (PALSAR-2) onboard Advanced Land Observing Satellite-2 (ALOS-2) of Japan Aerospace Exploration Agency (JAXA) on July 30, 2015. The ALOS-2/PALSAR-2 satellite image was originally produced by JAXA (http://en.alos-pasco.com/). The observed inundated areas for the 2016 flood event were from the Moderate Resolution Imaging Spectroradiometer (MODIS) flood inundation product of the National Aeronautics and Space Administration (NASA) of United States of America (NASA experimental product). The MODIS data were downloaded from Dartmouth Flood Observatory, University of Colorado (http://floodobservatory.colorado.edu/Myanmar.Html). The data sources are summarized in Table 1.

**Table 1:** Data sets and data sources.

Data Sets	Sources
Topography data: 15 seconds (approximately 450m) DEM, flow accumulation, and flow direction	USGS HydroShed (https://hydrosheds.cr.usgs.gov/datadownload.php?reqdata=15demg)
Rainfall and discharge: April to August of 2011, 2015 and 2016	Department of Meteorology and Hydrology, Mynamar (see S1 Dataset)
River cross section: Panhlaing River	River cross-section maps were from the pinted reports of Irrigation and Water Utilization Management Department, Mynamar (https://www.moali.gov.mm/en/content/irrigation-and-water-utilization-management-department). These printed maps were used to estimate the routing parameters required by the RRI model. The parameter values derived from the cross-section maps are summarized in S2 Dataset.
Land cover map 2008	Global Land Cover Nation Map Organization (https://github.com/globalmaps/gm_lc_v3)
Soil map	FAO UNESCO digitized soil map of the world (http://www.fao.org/soils-portal/soil-survey/soil-maps-and-databases/faounesco-soil-map-of-the-world/en/)
Digitized shapefile (e.g. rivers, fishery ponds, etc.)	Myanmar topographic survey map (2008 edition) (http://www.surveydepartment.gov.mm/eng/)
Satellite image (ALOS-2/PALSAR-2) captured on July 30, 2015	Sentinel Asia, JAXA (http://en.alos-pasco.com/)
MODIS flood inundation image (July 26 to Aug 8, 2016)	NASA experimental science product (http://floodobservatory.colorado.edu/Events/2016Myanmar4365/2016Myanmar4365.html)

### Model description

RRI is a two-dimensional coupled hydrological and inundation model, which include three key components: a rainfall-runoff model, a river routing model, and a flood inundation model. The RRI model was developed by the International Center for Water Hazard and Risk Management under the auspices of UNESCO (ICHARM). It can simultaneously simulate the rainfall-runoff processes and inundation [25]. Rainfall-Runoff Model performs the simulations of runoff generation, runoff concentration, and river streamflow discharge with precipitation as input. In this model, the River Routing Model (1-dimensional unsteady flow model) is used to model the propagation of flood wave along open channels. The geometry is assumed as a rectangle when information on river cross-section is not available. The river width and depth are estimated using the following equations according to the upstream contributing area in km^2^ [25]:

$$W=C_{w}A^{S_{w}}$$ (1)

and

$$D=C_{d}A^{S_{d}},$$ (2)

where W is width (m); D is depth (m); A is area (km^2^); *C*_*w*_ and *S*_*w*_ are width parameters; and *C*_*d*_ and *S*_*d*_ are depth parameters. Parameter values for routing are summarized in S2 Dataset.

A flood inundation model, *i*.*e*., a 2-dimensional unsteady flow model, is used for simulating flooded water spreading on the floodplains with inflow discharge [25]. The governing equations for the 2-dimensional unsteady flow model include the mass balance (Eq 3) and momentum equations (Eqs 4 and 5):

$$\frac{\partial h}{\partial t}+\frac{\partial q_{x}}{\partial x}+\frac{\partial q_{y}}{\partial t}=r,$$ (3)

$$\frac{\partial q_{x}}{\partial t}+\frac{\partial uq_{x}}{\partial x}+\frac{\partial vq_{x}}{\partial y}=-gh\frac{\partial H}{\partial x}-\frac{\tau_{x}}{\rho_{w}}$$ (4)

and

$$\frac{\partial q_{x}}{\partial t}+\frac{\partial uq_{x}}{\partial x}+\frac{\partial vq_{x}}{\partial y}=-gh\frac{\partial H}{\partial x}-\frac{\tau_{y}}{\rho_{w}},$$ (5)

where h is the water level from the local surface; *q*_*x*_ and *q*_*y*_ are the unit width discharges in *x* and *y* directions, respectively; *u* and *v* are the flow velocities in x and y directions, respectively; r is the rainfall intensity; H is the water level from the datum; *ρ*_*w*_ is the density of water; g is the gravitational acceleration; and *τ*_*x*_ and *τ*_*y*_ are the shear stresses in *x* and *y* directions, respectively.

Infiltration is an important hydrological process than largely influences runoff generation. Infiltration parameters can vary with different rainfall intensity, soil profile, and topography across the study area. It is considered that the Green-Ampt infiltration model is appropriate to model the vertical infiltration process [26, 27] as our study area is a flat and lowland area. The Green-Ampt infiltration model is a simplified physical model and based on the Richard equation. It relates the rate of infiltration to measurable soil properties such as the porosity, hydraulic conductivity, and soil water content of a particular soil column. The following equation is used to calculate the infiltration losses [25, 26]:

$$f=k_{v}[1+\frac{(\emptyset -\theta_{i})S_{f}}{F}],$$ (6)

where *f* is infiltration rate (mm/hr); *k*_*v*_ is the hydraulic conductivity (m s^-1^); *F* is the total volume of infiltration (m); *S*_*f*_ is the wetting front soil suction head (m); and (∅ − *θ*_*i*_) is the soil moisture deficit (m).

In RRI, each computational grid cell on the river has not only surface hydrological model and groundwater analysis model but also river channel model. The RRI model describes landform with DEM. The depth of permeable layer is one of the important parameters for RRI model. Before reaching saturation in soil layer, the model simulates water movement using Darcy’s Law in which phreatic water flows in lateral direction according to head differential of groundwater when rainfall penetrates into the ground. After soil is saturated, the model simulates water movement using a diffusion wave model and rainfall water starts flowing on the surface. In the lowland area, rainfall penetrates in the vertical direction. In this case, groundwater flow in the lateral direction is negligible because water head of groundwater is almost the same [28].

### Procedure of the RRI model simulation

Ground gauge rainfall data were prepared as the input data of the RRI simulation using the Thiessen polygon method. The RRI Model is equipped with a DEM adjustment tool, which is essential to avoid unrealistic condition and can do necessary modification of the original DEM based on the flow direction [29]. The model also requires information on the locations of river channels and the geometries of cross-sections. We used flow accumulation data sets included in the HydroSHEDS dataset. Grid cells with more than twenty grid cells were confirmed to have a river channel based on visual checking with Google Earth in this study [11, 27].

The river routing equations (width (m) = 5.0A^0.35^, depth (m) = 0.95A^0.2^) are used as default settings for deriving river channel widths and depths, where A is the upstream contributing area in km^2^ (Eqs 1 and 2). The channel geometry is assumed as a rectangle in the model in which the river width, depth, and embankment height were considered [27, 30]. In this study, the assumption of the river width is based on Google Earth images [27, 31] and the depth on the local survey data of the Department of Meteorology and Hydrology for Ayeyarwady River. The river width, depth and embankment height for the Panhlaing River, a tributary of Ayeyarwady River, are supported by the IWUMD Office at the Nyaungdon station [8]. Daily rainfall data from ten rainfall stations are used as input data for model runs (Fig 1). After parameterization, model is set up for the simulation. The output results from the model have flooded inundation depths, river discharges, and water levels at each grid cell. Observed discharge is not available within the study area due to lack of observation station. However, there is an upstream streamflow gauging station (*i*.*e*., Zalun) is located right on the upper boundary of the study area (Fig 1).

**Fig 4:**
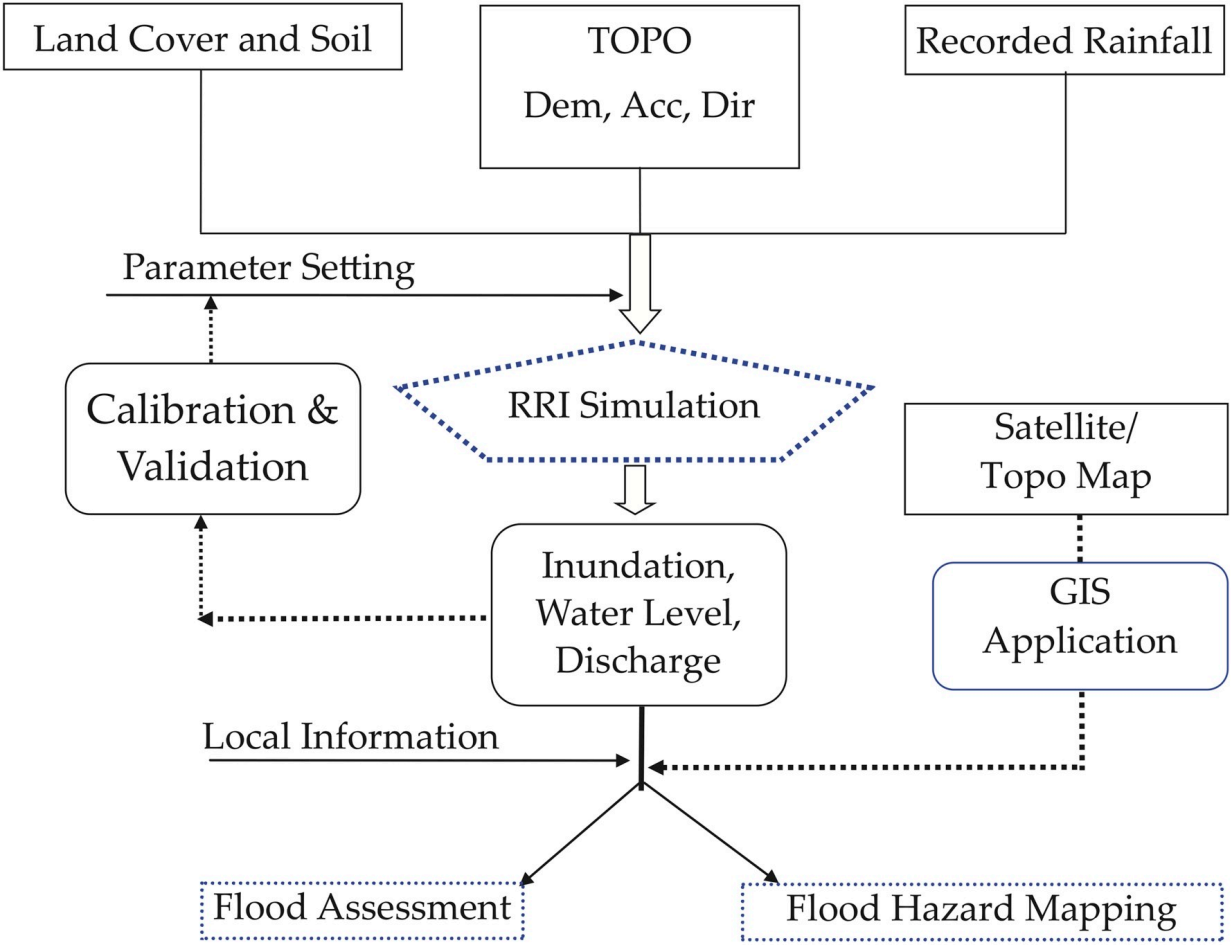
The flowchart of model simulation used in this study.

The procedure of model simulation is shown in Fig 4, including data processing, model simulation, model calibration, validation of modeled streamflow and inundation area, and flood hazards mapping and assessment. Finally, we chose the simulations of the 2011, 2015, and 2016 flood events to produce a flood hazard map for improving the flood assessment and hazard mapping.

### Model calibration and validation

Over the past decade, NDT was affected by the devastating flood hazards in 2011, 2015, and 2016. Although the study area has already experienced seasonal floods almost every year, the local flood hazard map and proper flood assessment are still lacking. This highlights a large knowledge and technical gap in the development of flood protection measures and flood risk management in this region.

Local surveying and investigation of hydrological information are very few in the study area, except the Panhlaing River. The cross-sectional profile for Ayeyarwady River and Bawle River are insufficient to set up the geometry data such as river width and depth. Considering the data limitation [15, 27, 32, 33], we chose the observed streamflow of the 2011 flood event at the upstream Zalun station, which is the discharge measuring station (see Fig 1) [11, 33], to calibrate the RRI model to achieve a better performance. The observed streamflow for the 2015 and 2016 flood events at the Zalun station are then used to validate the performance of modeled streamflow by the calibrated RRI model.

To further evaluate the robustness of the model calibration, we applied a cross-calibration to assess the model performance. In other words, we first calibrated the RRI model at the Zalun station using the 2016 flood event and then validated the model performance using the 2011 and 2015 flood events.

For the performance of inundation prediction by the RRI model, we obtained two sets of satellite remote sensing inundation images. One is the ALOS-2/PALSAR-2 inundation image observed on July 30, 2015 provided by JAXA (see Table 1), which is used to evaluate the model results for the 2015 flood event. The other one is the 14-days composite MODIS flood inundation product (26 July to 8 Aug, 2016) provided by NASA (see Table 1)[32, 34–36].

To evaluate the performance of modeled streamflow, we compared the modeled streamflow for the three flood events with the observed counterparts at the upstream Zalun station. Statistical metrics including Pearson’s coefficient of determination (R^2^) and Nash-Sutcliffe model efficiency coefficient (NSE) are used to evaluate the model performance:

$$R^{2}=\frac{{\left[\sum_{i=1}^{n}\left(\left(Q_{obs}(i)-\overset{-}{Q_{obs}})\times (Q_{sim}(i)-\overset{-}{Q_{sim}}\right)\right)\right]}^{2}}{\sum_{i=1}^{n}{\left(Q_{obs}(i)-\overset{-}{Q_{obs}}\right)}^{2}\times \sum_{i=1}^{n}{\left(Q_{sim}(i)-\overset{-}{Q_{sim}}\right)}^{2}}$$ (7)

and

$$NSE=1-\frac{\sum_{i=1}^{n}{\left(Q_{sim}(i)-Q_{obs}(i)\right)}^{2}}{\sum_{i=1}^{n}{\left(Q_{obs}(i)-\overset{-}{Q_{obs}}\right)}^{2}},$$ (8)

where Q_obs_(i) and Q_sim_(i) are the observed and simulated streamflow discharges at time *i*, respectively; $\overset{-}{Q_{obs}}$ and $\overset{-}{Q_{sim}}$ are the arithmetic means of observed and simulated streamflow discharges, respectively;

To evaluate the performance of inundation predictions by the RRI model, we followed the previous studies [27, 37–41] to conduct a comprehensive evaluation by comparing the satellite observations and model predictions on a cell-by-cell basis. The validation statistical metrics include flood area index (FAI) [42], accuracy, bias score, probability of detection (hit rate), false alarm ratio, probability of false detection, success index:

$$FAI=\frac{M1D1}{M1D1+M1D0+M0D1},$$ (9)

$$Accuracy=\frac{hits+correct\;negatives}{total},$$ (10)

$$Bias\;score=\frac{hits+false\;alarms}{hits+misses},$$ (11)

$$Probability\;of\;detection\;(hit\;rate)=\frac{hits}{hits+misses},$$ (12)

$$False\;alarm\;ratio=\frac{false\;alarms}{hits+false\;alarms},$$ (13)

$$Probability\;of\;false\;detection(false\;alarm\;rate)=\frac{false\;alarms}{correct\;negatives+false\;alarms},$$ (14)

and

$$Success\;index=\frac{1}{2}\;(\frac{hits}{hits+misses}+\frac{correct\;negatives}{correct\;negatives+false\;alarms}),$$ (15)

where M1D1 is the number of grid cells correctly predicted as flooded by model, *i*.*e*., the number of hits; M1D0 is the number of cells flooded in the prediction but observed as dry in the observation, *i*.*e*., the number of false alarms; and M0D1 is the predicted dry area but observed as wet area, *i*.*e*., the number of misses; the number of correct negatives are the number of cells predicted and observed as dry.

### Flood hazard mapping

The flood hazard mapping involves three steps: (1) identify the basic issues and conditions, (2) collect necessary data needed by flood simulation and inundation mapping, and (3) conduct flood hazard simulation and mapping. Circumstances of past flood events, potential extents of flood areas and flood depths in flood inundation maps are identified by stakeholders of the local area. Generally, flood hazard is defined as the probability of potentially damaging flood situations in a given area and within a specified time horizon [43]. A flood hazard map is map showing flood extents, water depths, key landmarks, and other base information for a flood hazard with a given return period [43]. To produce the flood hazard map, we collected the required data such as hydrological data, inundation conditions, disaster prevention activities and other relevant information from the NDT local government and the other related organizations in this study. Due to data limitation, there are no available long-term rainfall and stream flow data record in this study area. Therefore, we cannot directly derive or design a flood hazard with a given return period. Considering that the 2015 and 2016 flood events are the reported two largest flood events in recent 30 years [8]. We first applied the RRI model to simulate the flood depths and inundation areas for the two largest flood events. We then computed the average flood depths and extents of the two flood events to produce a flood hazard map that approximates the flood map of a flood event with about 30-year return period. The average flood depths were converted to six levels of flood risks: very low (depth≤0.2m), low (0.2m<depth≤0.5m), medium (0.5m<depth≤1.0m), very high (1.0m<depth≤2.0m), and extremely high (depth>2.0m). Regarding the landmarks and other base information, MIMU provided the locations of the city and villages and the boundary shape files such as township, districts, region, and country. The required shape files (e.g. rivers, tributaries, built-up areas, ponds, swamps, roads, etc.) were digitized from the Myanmar Topography Map 2008 edition. However, the locations of evacuation centers, the other public infrastructures, and information on early warning systems and emergency are unfortunately unavailable to be included in this developed flood hazard map due to data limitation.

## Results and discussion

### Results of model calibration and validation

**Fig 5:**
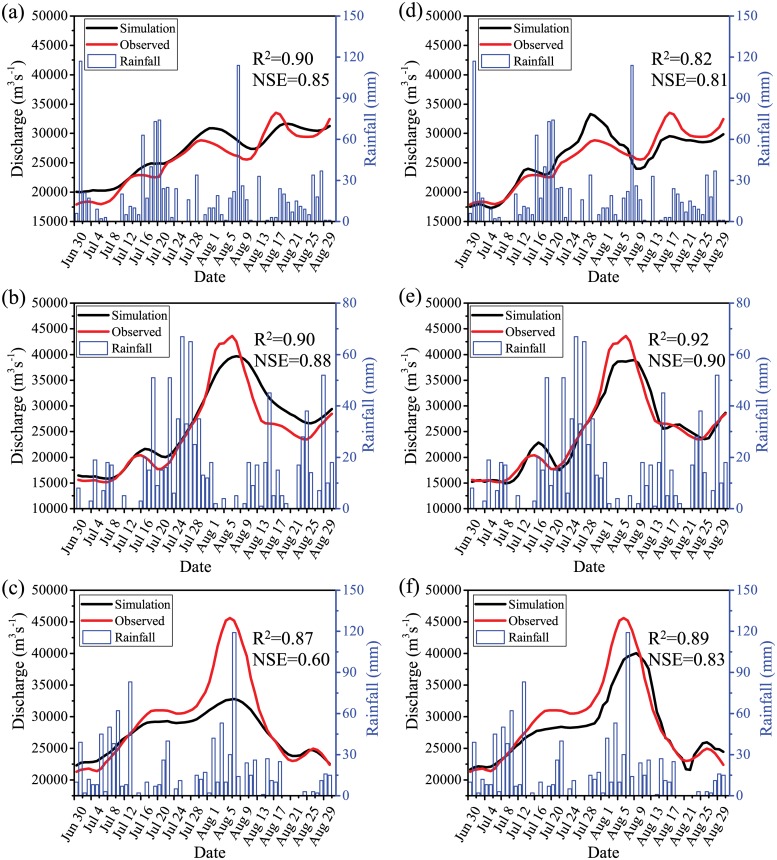
Comparisons of the simulated and observed discharges for the (a, d) 2011, (b, e) 2015, and (c, f) 2016 flood events at the Zalun station; (a)-(c) show the model results calibrated against the 2011 flood event, while (d)-(f) are the model results calibrated against the 2016 flood event, *i*.*e*., the cross-calibration results.

After calibration, the modeled streamflow discharges at the Zalun station matches well with the observations indicated by high *R*^2^ (0.90) and *NSE* (0.85) values (Fig 5a). The RRI model is generally able to capture the temporal variablity of the streamflow processes (Fig 5a). Certainly, there is still some uncertainty in the simulation. It can be seen that the simulated flow has higher values than the observed flow on the rising limb of the 2011 flood event (Fig 5a). In terms of the flood peaks, the simulated results overestimated the first two peaks (July) and underestimated the third peak (August) (Fig 5a). Despite the uncertainty, the calibration still gained an overall satisfactory result.

Regarding the validation results, the RRI model also shows a generally good agreement with the observations for the 2015 and 2016 flood events in terms of the statistical metrics (*R*^2^ = 0.90 and *NSE* = 0.88 for the 2015 flood event; and *R*^2^ = 0.87 and *NSE* = 0.60 for the 2016 flood event) (Fig 5b and 5c). The simulated results can generally capture the timings of flood peaks despite that the model simulations tend to underestimate the flood peaks (Fig 5b and 5c). In addition, it is clear that the RRI model has a better performance for the 2015 flood event than the 2016 flood event. These differential performances may result from the uncertainty in the model initial conditions and/or driving data. Comparing to the calibration result (Fig 5a), the validation results have a similar level of performance (Fig 5b and 5c), indicating that the calibrated model is able to provide consistent predicative capacity, at least for the periods close to the calibration period.

The cross-calidation shows similar results comparing to the original calibration. It is not surprise that the model simulation for the 2016 flood event that is calibrated against the 2016 flood event, *i*.*e*., the cross-calibrated simulation for the 2016 event (Fig 5f), shows a slightly better result than the model simulation calibrated against the 2011 flood event (Fig 5c) in terms of both the *R*^2^ and *NSE* metrics. On the contrary, the simulation for the 2011 flood event that is calibrated against the 2011 event (Fig 5a) shows a slightly better result than the cross-calibrated simulation for the 2011 event (Fig 5d). Overall, performance of the originally calibrated model is comparable to that of the cross-calibrated model in terms of the *R*^2^ and *NSE* metrics. The *R*^2^ and *NSE* values for the original calibrated model range between 0.87 and 0.90 with a mean of 0.89 and between 0.60 and 0.88 with a mean of 0.78, respectively (Fig 5a–5c). Similarly, the *R*^2^ and *NSE* values for the cross-calibrated model vary between 0.82 and 0.92 with a mean of 0.88 and between 0.82 and 0.92 with a mean of 0.85, respectively (Fig 5d–5f). The sligtly different model performance resulted from different calibration procedures indicate that calibration or uncertainty in the model parameters indeed can introduce certain uncertainty in the model simulations. However, the model performance through the original calibration (*i*.*e*., calibration using the 2011 flood event) is overall comparable to the performance resulted from the cross-calibration (*i*.*e*., calibration using the 2016 flood event). These results suggest that the calibration using the 2011 flood event is robust and can achieve a similar level of performance as the cross-calibration does (Fig 5).

**Fig 6:**
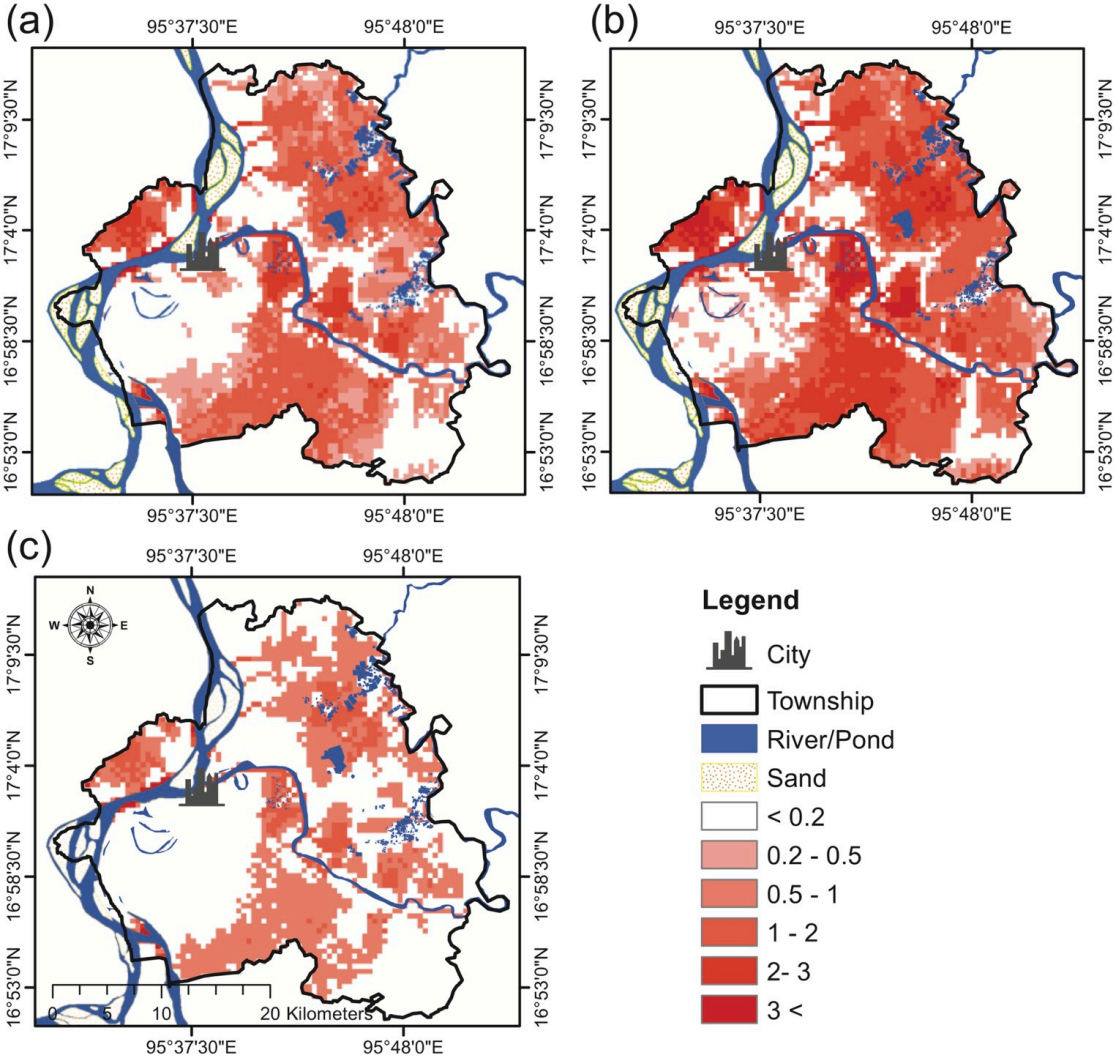
Model-simulated potential flood inundated areas for the (a) 2011 and (b) 2015 (c) 2016 flood events.

**Fig 7:**
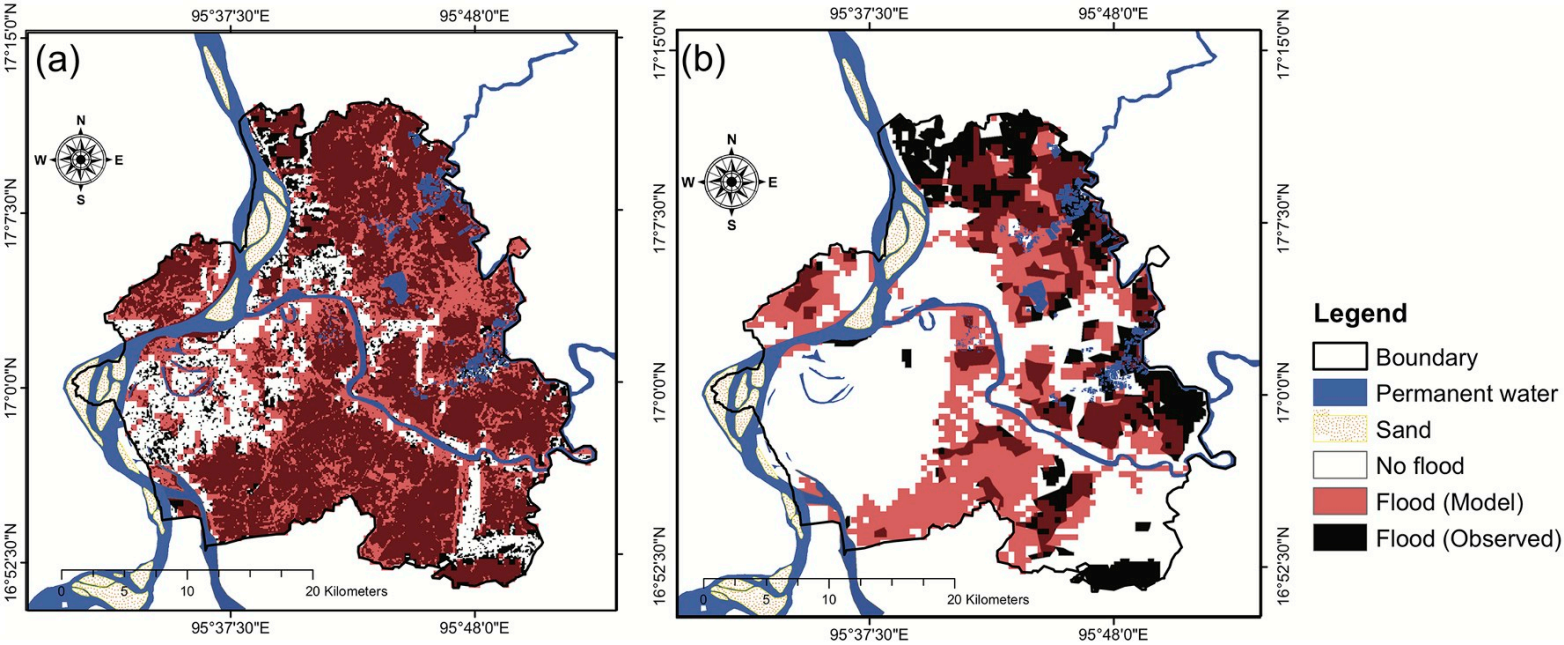
Comparison of flood extent areas: (a) model simulation and observed (MSI) for the 2015 flood event and (b) model simulation and observed (MODIS) for the 2016 flood event.

Furthermore, we also plotted the simulated inundated areas for the 2011 (Fig 6a), 2015 (Fig 6b) and 2016 (Fig 6c) flood events. The satellite-detected flood areas for the 2015 and 2016 flood events are also shown in Fig 7 as a means to evaluate the inundation model performance. The 2015 flood event apparently inundates more areas than the 2011 and 2016 flood events (Fig 6) because the 2015 flood event is a larger flood event. This is consistent with the recorded discharge (Figs 3b and 5). Apparently, the two flood events have many overlapping inundated areas, of which most are the flattened areas with low altitude and suffer frequent flood hazards (Fig 6).

The simulated potential flood areas stratified by water depth for the two flood events are summarized in Table 2. By comparing the potential flooded areas, the 2015 flood event has the highest impact in terms of the inundation area. During the 2015 flood event, 76% of the township is simulated to be flooded with a water depth of 1 m or above. In contrast, 63% and 38% of the township are predicted to be flooded by the similar level of water depth for the 2011 and 2016 events, respectively (Table 2). As shown in Fig 6, floodwater depth can reach as high as 3 m or above near the riverbanks and ponds. The flooded areas with a depth of 2–3 m are mainly fisheries and swamps, while these areas with a floodwater depth of 1 m or below are mainly these flood-susceptible urban areas and villages.

**Table 2:** Potential flood inundated area with different water depth in three flooded years.

Water depth (m)	2011		2015		2016	
	Area (km^2^)	%	Area (km^2^)	%	Area (km^2^)	%
0–0.5	74.12	14	70.27	11	111.98	33
0.5–1.0	121.50	23	82.22	13	97.40	29
1.0–2.0	244.22	46	263.66	42	107.33	32
2.0–3.0	70.27	13	167.87	27	20.25	6
> 3.0	19.24	4	41.51	7	0	0

For the 2015 flood event, the flooded area in the model simulation is 69% (619 km^2^) of the township area (~899 km^2^), while the flooded area detected by the ALOS-2/PALSAR-2 is 54% of the study area (488 km^2^) (Fig 7a). The overlapped areas between the model and ALOS-2/PALSAR-2 inundation results are 432 km^2^, *i*.*e*., 89% of the satellite-observed flooded areas (Fig 7a). Overall, the modeled simulation result for the 2015 event agrees reasonably well with the satellite observation indicated by a hit rate of 89% and an accuracy of 70% (Table 3). These results indicate that the RRI model is able to realistically predict the flooded areas. It is worth noting that the RRI tends to overestimate the inundated area indicated by a false alarm ratio of 30%, a false alarm rate of 57%, and a bias score of 1.27 (Table 3; Fig 7a). As a result, the model prediction for the 2015 flood event ends up a success index of 66%. For a data-sparse region, this result is satisfactory.

**Table 3:** Summary of the modeled and satellite-observed inundation results and the model performance metrics.

Values (km^2^)	2015	2016	Performance metrics	2015	2016	(2015+2016)
M1D1	432.31	152.48	FAI	0.64	0.43	0.57
M1D0	186.57	149.65	Accuracy	0.70	0.72	0.71
M0D1	55.75	48.60	Bias score	1.27	1.50	1.34
Hits	432.31	152.48	Hit rate	0.89	0.76	0.85
Misses	55.75	48.60	False alarm ratio	0.30	0.50	0.37
Correct negatives	141.36	368.96	False alarm rate	0.57	0.29	0.40
False alarms	186.57	149.65	Success index	0.66	0.73	0.73

For the 2016 flood event, model prediction shows a slightly better performance than the 2015 flood event. The flooded area in the model simulation is 34% (302 km^2^) of the study area, while the flooded area detected by the MODIS images is 22% (201 km^2^) (Fig 7b). The overlapped areas between the model and MODIS inundation results are 152 km^2^, *i*.*e*., 76% of the satellite-observed flooded areas (Fig 7b). Relative to the prediction results for the 2015 flood event, the prediction results for the 2016 flood event have a higher accuracy (72%) and success index (73%) and a lower false alarm rate (29%) but have a lower hit rate (76%) (Table 3). Similar to the results for the 2015 flood event, these results for the 2016 flood event also tends to overestimate the inundated areas (Fig 7).

When combining the 2015 and 2016 events together, the resultant performance metrics are similar to those of the two individual events (Table 2). Overall, the results are encouraging indicated by high hit rate, accuracy, and success index despite that the model tends to overestimate the inundation extent (Table 3). There are a couple of causes that can explain the overestimation in the model results for the 2015 and 2016 flood event. Both ALOS-2/PALSAR-2 and MODIS images have a revisit time longer than a few days, making them impossible to continously observing a given region. The ALOS-2/PALSAR-2 uses a synthetic aperture radar (SAR) to observe the ground and has a capability to penetrate the clouds. However, its revisit time is about 14 days. The MODIS inundation images represent a 14-days composite that cannot observe our study area continuously. Therefore, it is reasonable to infer that the ALOS-2/PALSAR-2 and MODIS products can underestimate the inundation extent of a large flood event that lasts for more than a couple of days. Moreover, the MODIS images are based on optical and near-infrared remote sensing and can be easily contaminated by clouds, which can further reducing the chances to capture the inundated areas. However, unfortunately, the study area is a data-scarce region. We could not find a better way based on the other modeling approach or ground survey to discern flood areas and determine the flood inundation depth in this study area. Nevertheless, the model calibration and validation suggest that the model simulation results through this study give satisfactory information. Furthermore, the RRI model and its predictive capability for flood and inundation is very valuable for achieving the stated objectives and filling the knowledge and information gap in enhancing the flood risk mitigation and management in Nyaungdon Area, Myanmar.

### Flood hazard mapping

**Fig 8:**
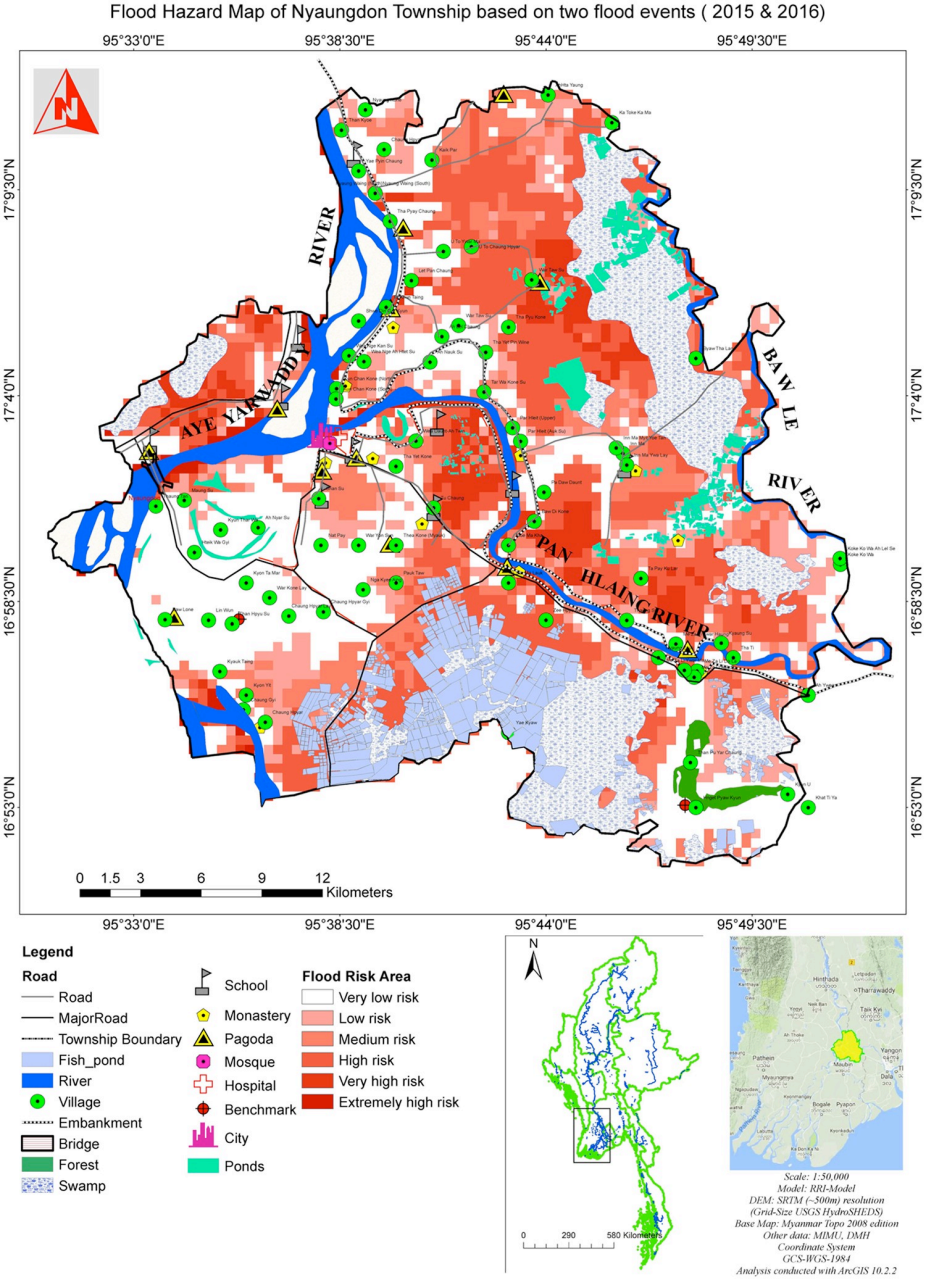
Flood hazard map of Nyaungdon Township based on the composite results of the 2015 and 2016 flood events, which corresponds to a flood event with about 30-year return period.

The flood hazard map is produced based on the composite results of the 2015 and 2016 flood events to approximate a 30-year flood event and shown in Fig 8. In this hazard map, important locations and facilities such as pagodas, monasteries, mosques, hospitals, schools and benchmarks are collected from the 2008 edition of Myanmar Topography Map (Fig 8). Generally, most of the areas impacted by floods are these areas close to rivers, ponds, swamps, and fisheries (Fig 8). Clearly, the floods can also impact many of the dowelling communities. For example, about 31% of the 140 villages in this region are inundated to some extent according to the inundation simulation (Fig 8). The major roads are also exposed to the flood hazards (Fig 8). Many important locations and facilities are impacted or threatened by the flood, including six schools, six monasteries, seven pagodas and one mosque (Fig 8).

On the positive side, the river embankment in Nyaungdon Area is able to prevent many urban areas from flooding (Fig 8). For example, a large area surrounding the city located in the west of the study area is clearly protected by the river embankments and has a low risk of flooding. The flood hazard map also indicates that the protection of the embankments along the Panhlaing River located in the southeast of the study area is limited (Fig 8). This is mainly due to the relatively low elevation in the floodplains along the Panhlaing River. In the future, implementing enhanced flood control structures and measures to relieve waterlogging should be considered in these areas for preventing inundation. The western part of the study area has a much lower risk of flooding than the other parts. This region should be a safe place to house the future important facilities. In summary, the derived flood hazard map has identified the flood risks across Nyaungdon Area and is very valuable for future flood management and design of flood control structures in this region.

## Conclusions

In this study, the coupled hydrological and inundation model (RRI) was applied for flood simulation and inundation mapping in data-scarce Nyaungdon Township, Myanmar. The calibration and validation results of the streamflow generally show good agreement with the observations in terms of both Pearson’s coefficient of determination and Nash-Sutcliffe efficiency coefficient. The RRI model can realistically capture the observed hydrographs and the timings of flood peaks. The modeled inundation areas for the 2015 and 2016 flood events can capture 89% and 76% of the flooded areas observed by satellites, respectively. Considering the data limitation in this data-scarce region, the distributed RRI coupled hydrological and inundation model plays an important tool for assessing flood risks and mapping the flood hazards. This study not only provides a valuable evaluation of the RRI model but also demonstrates a useful case study for applying the coupled hydrological and inundation model for flood hazard mapping. In the future, additional river survey data, high-resolution satellite images, and aerial photos should be used for calibrating the RRI model and improving the accuracy of flood hazard mapping. More hydrometeorological observation stations are advocated to be installed in Nyaungdon and its surrounding area to provide first-hand hydrological information.

## Supporting information

S1 Dataset Observed streamflow data at the Zalun station, river stage data at the Nyaungdon station, and basin-averaged rainfall data. (XLSX)

S2 Dataset Values of the RRI model parameters used in this study. (DOCX)

S3 Dataset Digitized shape files by this study. (ZIP)
