# Supplementary material for: Flood hazard mapping and assessment in data-scarce Nyaungdon area, Myanmar
Source: PLoS One. 2019 Nov 26;14(11):e0224558. doi: 10.1371/journal.pone.0224558 (PMC6879136; doi:10.1371/journal.pone.0224558)
Supplement: S2 Dataset — (DOCX) [file pone.0224558.s002.docx]

River routing model and parameters

The river width and depth are approximately by using the following functions according to the upstream contributing area in km^2^ :

$W=C_{w}A^{S_{w}}$

$D=C_{d}A^{S_{d}}$

where W is width (m), D is depth (m), A is area (km^2^), C_w_ and S_w_ are width parameters, and C_d_ and S_d_ are depth parameters.

In this paper, Panhlaing River cross-section data (river width and depth) were used to adjust the river routing parameters. The fixed parameter values are:

$c_{w}$ = 5.0; $s_{w}$ = 3.5; $c_{d}$ = 0.95; $s_{d}$ = 0.2.
